# Analysis of Effective Interconnectivity of DegraPol-foams Designed for Negative Pressure Wound Therapy

**DOI:** 10.3390/ma2010292

**Published:** 2009-03-25

**Authors:** Vincent Milleret, Anne Greet Bittermann, Dieter Mayer, Heike Hall

**Affiliations:** 1 Department of Materials, ETH Zurich, Zurich, Switzerland; E-Mail: vincent.milleret@mat.ethz.ch; 2 ZMB, Center for Microscopy and Imaging Analysis, University of Zurich, Switzerland; E-Mail: agb@access.unizh.ch; 3 Cardiovascular Surgery, University Hospital Zurich, Zurich, Switzerland; E-Mail: dieter.mayer@usz.ch

**Keywords:** Degrapol^®^, polymer foams, pore interconnectivity, Negative Pressure Wound Therapy (NPWT)

## Abstract

Many wounds heal slowly and are difficult to manage. Therefore Negative Pressure Wound Therapy (NPWT) was developed where polymer foams are applied and a defined negative pressure removes wound fluid, reduces bacterial burden and increases the formation of granulation tissue. Although NPWT is used successfully, its mechanisms are not well understood. In particular, different NPWT dressings were never compared. Here a poly-ester urethane Degrapol^®^ (DP)-foam was produced and compared with commercially available dressings (polyurethane-based and polyvinyl-alcohol-based) in terms of apparent pore sizes, swelling and effective interconnectivity of foam pores. DP-foams contain relatively small interconnected pores; PU-foams showed large pore size and interconnectivity; whereas PVA-foams displayed heterogeneous and poorly interconnected pores. PVA-foams swelled by 40 %, whereas DP- and PU-foams remained almost without swelling. Effective interconnectivity was investigated by submitting fluorescent beads of 3, 20 and 45 μm diameter through the foams. DP- and PU-foams removed 70-90 % of all beads within 4 h, independent of the bead diameter or bead pre-adsorption with serum albumin. For PVA-foams albumin pre-adsorbed beads circulated longer, where 20 % of 3 μm and 10 % of 20 μm diameter beads circulated after 96 h. The studies indicate that efficient bead perfusion does not only depend on pore size and swelling capacity, but effective interconnectivity might also depend on chemical composition of the foam itself. In addition due to the efficient sieve-effect of the foams uptake of wound components *in vivo* might occur only for short time suggesting other mechanisms being decisive for success of NPWT.

## 1. Introduction 

Each year, approximately twenty million people around the world suffer from chronic wounds caused by diabetes (e.g. 7.0 million diabetic ulcers), circulatory problems and many other conditions such as surgical site infections that generate huge demands on the health care systems [1]. Negative Pressure Wound Therapy (NPWT) provides an efficient method which may reduce the length of treatment, speed up the clearance of infections and wound closure and reduce in-hospital days [2,3,4,5,6]. Therefore NPWT is a cost effective and versatile method for many applications as compared to conventional and advanced wound dressings [7,8,9]. NPWT applies a controlled level of negative pressure on wounds leading to accelerated debridement and promotion of healing in many different types of wounds [2,3,4,10,11,12]. The effects of NPWT include the fast removal of interstitial fluid through the negative pressure, decrease in local edema, increased blood flow and therefore decrease tissue bacterial loads. However, NPWT does not replace classic methods of wound care such as debridement and infection treatment. Moreover, the mechanical deformation of cells in and around the wound is thought to result in increased matrix synthesis, which ultimately leads to an improved wound healing [2,13,14].

Here poly-ester urethane Degrapol^®^ (DP)-foams [15,16,17] were produced and explored for their potential use as NPWT dressings. Structurally, DP-polymers are block co-polyester-urethanes containing two different polyester blocks: a rigid one that is crystallisable and an elastic block that is amorphous. Due to this structure, DP has highly elastomeric properties and can be produced to have different shapes such as foams, fibers, fleeces with adjustable mechanical characteristics [15,18,19], moreover DP was shown to be cyto- and hemocompatible suggesting its use as a medical polymer [17,20].

Currently used NPWT materials consist mainly of polyurethane (PU)- or polyvinyl alcohol (PVA)-foams (KCI Inc, [21,22]), that are placed on/into the wound site prior to application of negative pressure. Only very few studies report the use of alternative dressings such as an iodine-impregnated drapes [23,24]. PU-based foams consist of block co-polymers with hard and soft segments, where hard segments consist of urethane forming glassy or semi-crystalline domains [25]. Thermoplastic PUs are commonly used as implantable materials showing high mechanical strength, toughness, abrasion resistance and resistance to degradation in watery solution. PU-based materials are regarded as tissue tolerant and therefore often used for applications that have direct blood contact such as in NPWT [21,22,26,27].

Polyvinyl alcohol (PVA) is prepared by partial or complete hydrolysis of polyvinyl acetate to remove acetate groups and then polymerized. Polyvinyl alcohol has excellent film forming, emulsifying, and adhesive properties [28,29,30]. It has high tensile strength as well as flexibility. However these properties are dependent on humidity, such that under higher humidity conditions more water is absorbed. Water acts as a plasticiser reducing PVA’s tensile strength and making it softer, but increases its elongation and tear strength [31]. Therefore it has been clinically used to prevent post-operative adhesions [32,33] or as cartilage replacement [31,34].

The overall material characteristics of DP-, PU- and PVA-foams do not provide an explanation for the success of NPWT, therefore a comparative study to elucidate their functionality *in vitro* was performed. The aim of this study was to compare in an *in vitro* system DP-foams with commercially available PU- and PVA-foams (from KCI Inc., [14,22]) that are clinically used for NPWT. Swelling and pore sizes of DP-, PU and PVA foams were analyzed by scanning electron microscopy and the effective interconnectivity of the pores was analyzed by fluorescent bead perfusion experiments. Moreover, perfusion of differentially sized beads was analyzed with and without pre-adsorption of serum albumin to simulate more native conditions.

## 2. Results and Discussion

In this study DegraPol^®^ (DP)-foams were produced and their potential use as dressings for negative pressure wound therapy (NPWT) was explored. Morphology, swelling and effective interconnectivity of pores that is needed when negative pressure is applied through the foams to remove wound fluid, bacteria and cells during the therapy was compared between DP-foams and commercially available foams that are clinically used for NPWT in wound healing.

### 2.1. Morphological analysis and swelling of DP-, PU- and PVA-foams

**Figure 1:**
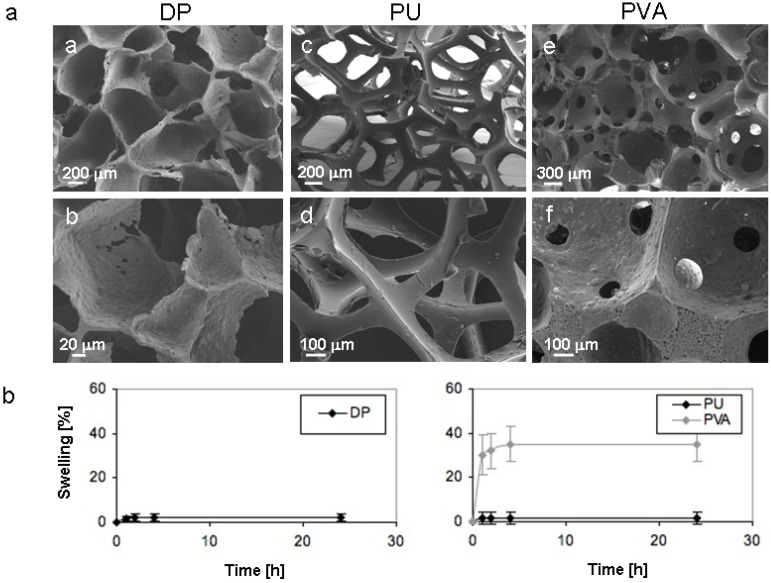
Morphological analysis and swelling of polymer foams.

In order to compare Degrapol^®^ (DP-) with PU- and PVA-foams, cross sections of all materials were analyzed by scanning electron microscopy (Figure 1a). DP-foams were porous having a homogeneous pore diameter of 160 ± 50 μm and the pores were well interconnected (a and b). PU-foams appeared highly porous with a mean pore diameter of 680 ± 140 μm. The pores seemed to be quite homogeneous in size and very much interconnected with each other, as the pores were lined only by slim connections resulting in an almost 3D-mesh-structure (c and d). In cross-sections of PVA-foams, the pores appeared variable in diameter (e and f). The mean diameter was 610 ± 420 μm, where pore diameters ranged from 100-1100 μm. The pores appeared much less interconnected with each other as compared to DP- and PU-foam pores and some pores even remained closed towards adjacent pores. After morphological characterization equilibrium swelling of the foams was compared (Figure 1b). The increase in diameter of DP-, PU- and PVA-foams was analyzed after swelling in either phosphate buffered saline (PBS) or simulated body fluid (SFB, not shown) at the indicated time points. No difference could be observed between the two solvents. The diameter of PVA-foams increased by ~ 41 ± 12 % showing increased softness as to be expected when PVA-foams are swollen in watery solution. In contrast DP- and PU-foams showed considerable less swelling (< 2 ± 3 %). Equilibrium swelling was reached for all types of foams after 4 h.

(a) DegraPol^®^ (DP-), PU- and PVA-samples have been analyzed by scanning electron microscopy (Figure a, - f). Samples were swollen, chemically fixed and critical point dried followed by sputtering with platinum. DP-foams demonstrate homogeneous and interconnected pores with small pore size. PU-foams display homogeneous pores with large diameter and high interconnectivity, whereas PVA-foams show heterogeneous pore sizes with different amount of pore interconnectivity. (b) DP-, PU- and PVA-foams were swollen to equilibrium in PBS. Shown is percentage of swelling for the increase in diameter after the indicated time points. Data represent means of 4 different samples per condition ± standard deviation. The values were normalized to the diameter prior to swelling (= 0 % swelling).

### 2.2. Determination of the effective interconnectivity of DP-, PU- and PVA-foams by bead perfusion experiments

**Figure 2:**
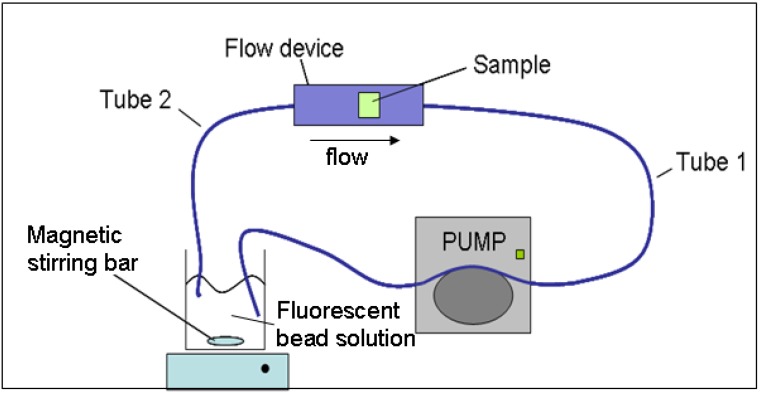
Experimental setup for perfusion experiments.

**Figure 3:**
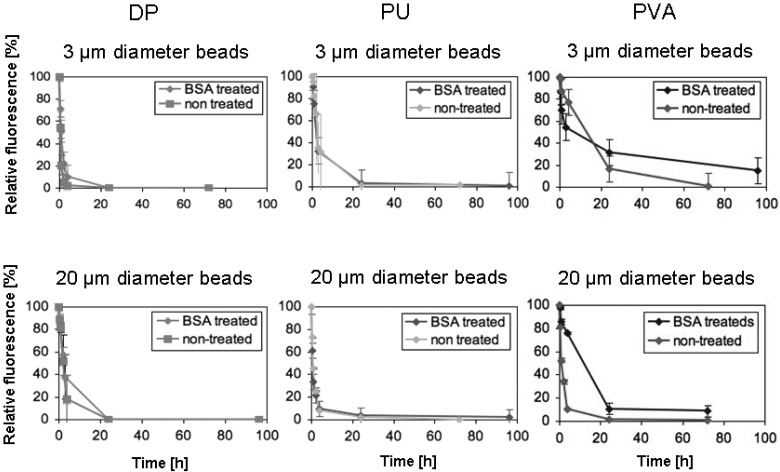
Effect of pre-adsorption with bovine serum albumin of fluorescent beads on perfusion time.

As foams when used for NPWT are exposed to a slight vacuum (usually between -80 and 125 mmHg) this study aimed at investigating the effective interconnectivity of the pores under flow conditions to simulate uptake of vacuum-removed body fluids, bacteria or cell debris. Removal of fluorescent beads from solution was determined as a measure for the uptake of beads by the foams. As evident from the SEM images and from swelling experiments, differences in pore diameter and interconnectivity might indicate differential perfusion with fluorescence beads. Fluorescent beads of different diameters were used for perfusion as they correspond roughly to the size of bacteria (3 μm), single cells (20 μm) and small cell clumps (45 μm) that need to pass through the foams while applied in NPWT. To analyze bead perfusion, a flow device was used that could harvest polymer cylinders with a diameter of 5 mm and 2-5 mm in length. The flow device was connected to a pump, enabling continuous fluorescent bead supply and a reservoir chamber where beads were stirred by a magnetic stirring bar such that they did not precipitate. A schematic of the closed-loop system is shown in Figure 2. In initial experiments fluorescent beads of 3 and 20 μm diameter were either used directly after rinsing extensively in PBS or after pre-adsorption with bovine serum albumin (BSA). The reason for this experiment was to determine whether pre-adsorption with albumin, the most frequent serum protein, would affect perfusion of the beads through the polymer foams (Figure 3). As the perfusion experiments were carried out in the presence of BSA, also the foams were pre-adsorbed thus simulating to a certain extent the native situation when the foams are exposed to blood and/or serum during their clinical application.

Schematic of the closed loop-system connecting a flow device (harbouring the sample to be analyzed) through silicone tubing to a pump and to a reservoir, containing the fluorescent bead solution. A magnetic stirrer inhibits precipitation of the beads.

The bead perfusion experiments showed that BSA-adsorbed and native beads performed very similarly in DP- and PU-foams, as 90 and 70 %, respectively, of the 3 μm diameter beads were entrapped after 4 h. Essentially all beads of 3 μm diameter were removed from solution after 24 h. 80-90 % of 20 μm diameter beads were adsorbed after 4 h for DP- and PU-foams, respectively, and all 20 μm diameter beads were removed from solution after 24 h. In contrast, for PVA-foams, BSA-pre-adsorption showed a difference for 3 and for 20 μm diameter beads, in that 3 μm diameter BSA-pre-adsorbed beads circulated considerably longer over the entire testing period of 96 h than non-treated beads. 20 % of the 3 μm diameter beads remained circulating even after 96 h. For 20 μm diameter beads this effect was significant as 90 % of BSA-pre-adsorbed beads were entrapped within the foams only after 24 h, whereas non-treated beads were removed from the solution already after 4 h. For all further studies BSA-pre-adsorbed beads were used as the presence of serum albumin has to be considered for *in vivo* applications. These experiments revealed that although DP- and PU-foams differed considerably in their pore size the effective interconnectivity for fluorescent beads did not reflect the morphological appearance. Fluorescent beads were taken up by DP- and PU-foams very efficiently within short time and this finding was almost independent from the bead diameter as well as from pre-incubation of beads and scaffold with serum albumin.

DP-, PU- and PVA-foams were perfused with 3 and 20 μm diameter fluorescent beads for 0 to 96 h, respectively. The beads were rinsed in PBS or pre-adsorbed with 2 % bovine serum albumin (BSA) prior to circulation through the foams. The values represent mean values ± standard deviation of 3 independent samples per condition where the relative fluorescence in (%) was determined at indicated time points.

Bead perfusion through DP-, PU- and PVA-foams was visualized microscopically as strongly fluorescent beads could be observed by yellow depositions on black PU- and white DP- or PVA-foams, respectively (Figures 4a, b and c). DP-, PU- and PVA-foams were perfused for 24 h with 3 and 20 μm diameter beads. DP-foams were analyzed from the side where the bead solution entered into the foam (influx), a cross section through the foam and on the opposite site where the solution left (outflux). Although DP-foams have by far the smallest pore size, 3 μm diameter beads distributed homogeneously throughout the entire DP-foam, as there was no visible difference between influx and outflux side. The cross section showed a homogeneous distribution as well. When 20 μm diameter beads were used a clear difference was observed between influx and outflux side as the beads accumulated at the influx side. The cross-section indicated the flow profile of the beads within the lumen of the DP-foam. In PU-foams it could clearly be seen that at the incoming site beads were accumulated and adsorbed to the struts of the foam as well as within the foam pores (Figure 2b). This was observed especially for 3 μm diameter beads. On the side of bead outflow, 3 μm diameter beads were also frequently found whereas 20 μm diameter beads were less visible. Surprisingly, within PVA-foams 3 μm diameter beads penetrated very well as similar amounts of fluorescent beads were visualized on both sides of the PVA-foam (Figure 4c). 20 μm diameter beads penetrated less through the foam as fewer beads were visible at the foam surface where the beads exited.

**Figure 4:**
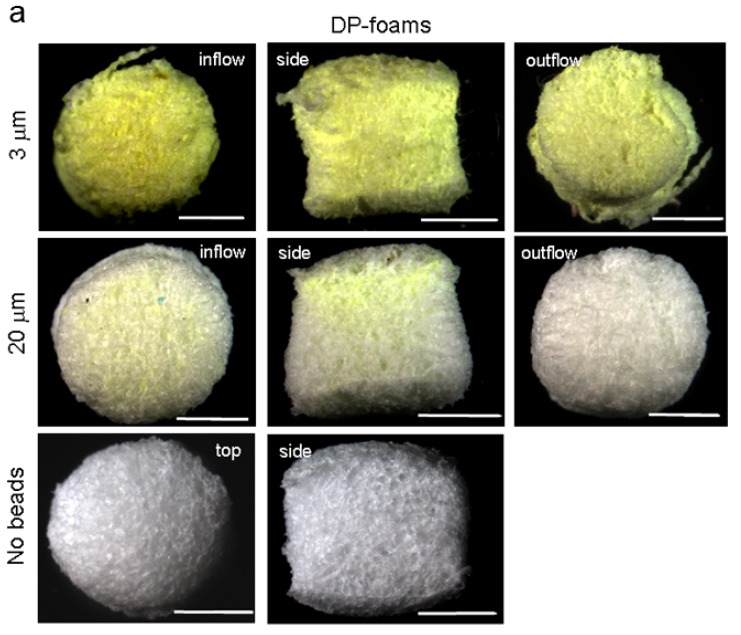
Perfusion of DP-, PU- and PVA-foams with BSA-pre-adsorbed and differentially-sized beads.

DP-foams were perfused with fluorescent beads of 3 and 20 μm diameter that roughly mimique the size of bacteria or single cells. The beads were pre-adsorbed with bovine serum albumin (BSA). Bead-perfused DP-foams were removed out of the flow device after 24 h and morphologically analyzed. The left images indicate foams that were exposed to the beads flowing into the foam coming from the reservoir container (inflow), whereas the right images display the foam surfaces of the opposite site of the foam (outflow). DP-foams were also shown as cross-section through the middle of the foam. The bottom two images show foams that were not exposed to any bead solution; left: top view, right: side view. The scale bar for all is 2 mm.

**Figure 4. Cont.**
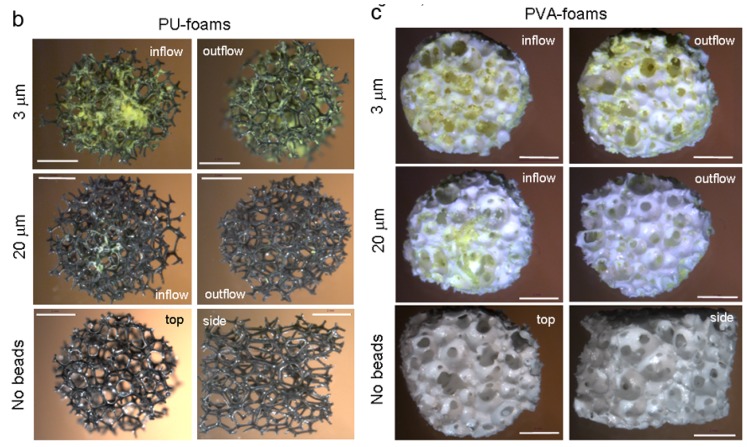
b and c) PU- and PVA-foams were perfused with fluorescent beads of 3 and 20 and analyzed as described for (a). The left images indicate foams that were exposed to the beads flowing into the foam coming from the reservoir container (inflow), whereas the right images display the foam surfaces of the opposite site of the foam (outflow). The bottom two images show foams that were not exposed to any bead solution; left: top view, right: side view. The scale bar for all is 2 mm.

**Figure:**
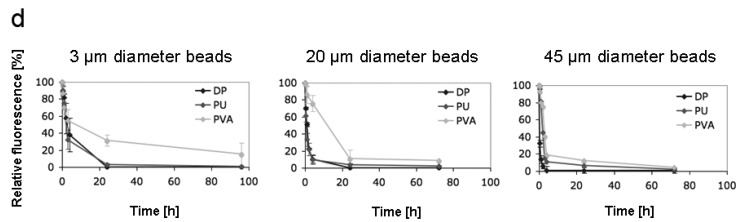
(d) demonstrates quantification of perfusion experiments using 3, 20 and 45 μm diameter beads. The beads were pre-adsorbed with bovine serum albumin (BSA) and the perfusion times were compared between DP-, PU- and PVA-foams for the given diameter of beads. The experiments indicate mean values ± standard deviations of three independent samples for each condition.

When bead perfusion experiments were quantified and compared, it could clearly be shown that DP-foams behave very similar to PU-foams (Figure 4d). For 3 μm diameter beads, 70 % of all beads were entrapped within the DP- and PU-foams after 4 h and essentially all beads were absorbed after 24 h; whereas in PVA-foams 60 % of the beads still permeated after 4 h and 20 % remained after 96 h. For 20 μm diameter beads 90 % of all beads were adsorbed in DP- and PU-foams after 4 h and almost all beads after 24 h. In PVA-foams 20 μm diameter beads circulated significantly longer and 10 % of the beads were still found circulating after 96 h. For 45 μm diameter beads, only little difference in perfusion time between the three foam materials was observed, as essentially all beads were entrapped within DP-foams after 4 h, 90 % of the beads in PU-foams after 4 h and 80 % of the beads after 4 h in PVU-foams.

The conclusion from these findings is that the pore size, swelling and interconnectivity of pores are not the only parameters decisive for effective bead penetration but also the chemistry of the foam, resulting in differential affinity between the beads and the polymer needs to be considered. Consistent with our findings would be that PU-foams induce very strong granulation tissue formation *in vivo* that often results in tight tissue ingrowth into the PU-foams during NPWT (personal communication D. M.). This is found less when PVA-foams were used. Other groups have explored PVA’s chemical characteristics when using PVA-films or foams in applications where tissue ingrowth is not desired such as to prevent postoperative adhesions [32,33].

Our study tries to dissect NPWT-efficiency into different aspects that contribute to its successful use in wound healing. Here we analyzed purely materials related properties being effective foam interconnectivity, swelling and uptake of small particles. The length of the treatment varies depending the wound type and size being usually between 2 and 15 days or longer with foam changes every 2-3 days [3,4,10]. During that time often an alternating schedule of negative pressure of -80 to -125 mmHg and normal pressure is applied to the wound that have been proven to be most efficient [35,36]. Two reasons might be thinkable why this is the most efficient way of vacuum application: one reason might be that NPWT dressings might clot less when alternating vacuum and non-vacuum phases are applied. Our study nicely showed that commercial DP-, PU- and PVA-foams uptake fluorescent beads very fast and that the beads accumulated within the pores of the foams. Therefore it might well be that alternating vacuum and non-vacuum phases might reduce the sieve-effect and therefore prolong the vacuum function of the NPWT dressings. Another reason might be that underlying cells are mechanically stimulated by alternating cycles of negative and normal pressure to form granulation tissue, secrete growth factors and collagens, form new blood vessels and start the healing processes [2,10,14]. This hypothesis has been analyzed comparatively between a finite element model of a wound to study cell deformation after application of strain and histological sections from tissues after NPWT [2]. The conclusion of their findings was that those differences in pressures and a mismatch between the elasticity of the wound and the NPWT-dressing result in mechanical stimulation of the wound that induces cell proliferation. Histological sections of wound biopsies reflected these findings showing increased tissue formation and vascularisation in high deformation areas [2,37]. Luckily a recent study demonstrates the development of a bioreactor type device that allows three-dimensional cell culture under subatmospheric pressure [13]. Moreover, a rodent *in vivo* model for NPWT was very recently established [14]. Therefore materials and biology-related questions that are relevant to explain the mechanisms of NPWT can be analyzed under more defined conditions in future studies.

## 3. Experimental Section

### 3.1. DP-foams

The polyester-urethane DP (trade name DegraPol^®^) was produced according to the procedure described by Lendlein *et al*. [38,39]. In brief: for the synthesis of the block copolymer, 40 wt % of poly(3-(*R*)-hydroxybutyrate)-co-(ε-caprolactone)-diol Mn = 2,660 and 60 wt % poly(ε-caprolactone)-diol were dissolved in 1.4-dioxane and dried by heating and refluxing the solvent over molecular sieves (pore size 0.4 nm) situated in a Soxhlet apparatus mounted onto the reaction vessel, until the water content was below 20 ppm. The reaction mixture was cooled to 83 °C before the stochiometric amount, with respect to the two diols, of diisocyanate (TMDI) was added. After about one day of reaction, three portions of dibutyltin dilaurate (20 ppm) were added within one day in order to reach molecular weights between 60 and 110 kDa. The polymer was precipitated in dry ice cooled methanol and subsequently purified via dissolution in chloroform and filtration over a silicagel 60 (Fluka) column. A second precipitation in dry ice cooled methanol ended the process. DP-foams were produced by a freeze-immersion precipitation technique. A solution of DP in dioxane as solvent (5 wt %) was prepared and cooled to 10°C for 2 h. The cold solution was poured into a stainless steel mold having the shape of the desired porous body. The solution in the mold was then cooled to -25 °C in a laboratory freezer and kept at this temperature overnight. Afterwards the solidified structure was taken out of the mold and air dried at room temperature in vacuum until complete removal of the dioxane. Cylinders of the polymer were punched out for perfusion experiments after swelling to equilibrium.

### 3.2. PU- and PVA-foams

Sterile black polyurethane foam dressings (V.A.C.^®^ GranuFoam^®^, 10 x 7.5 x 3.3 cm) and, polyvinyl-alcohol foam dressing (V.A.C.^®^ WhiteFoam, 10 x 7.5 x 1 cm) were purchased from the V.A.C. ^®^ T.R.A.C. ™ Therapy systems, KCI Medical Products, Wimborne, UK)**.** Cylinders of the polymer were punched out for perfusion experiments after swelling to equilibrium.

### 3.3. Swelling Experiments

In order to determine the increase in diameter and length of DP-, PU- and PVA-foams when exposed to phosphate buffered saline (PBS) or simulated body fluid (SFB), the foams were swollen to equilibrium. Swelling time and amount of swelling was determined for DP- and PU-foams of 5 mm in diameter and 5 mm length or 5 mm in diameter and 3 mm length. PVA-foams were analyzed using cylinders of 5 mm in diameter and 3 mm length or 5 mm in diameter and 2 mm in length. The samples were immersed in PBS (Sigma, P3688-10PAK) filled up to 1 L with double distilled water (Nanopure diamond, Barnstead) or SFB. SBF was prepared by mixing a “Ca^2+^ stock solution” and a “PO_4_^3-^ stock solution” in 1: 1 ratio prior to use. The compositions of those solutions were the following: Ca^2+^ stock solution (15.99 g NaCl; 0.4474 g KCl; 0.6099 g MgCl_2_ x 6 H_2_O; 1.0955 g CaCl_2_ x 6 H2O); PO_4_^3-^ stock solution (0.3222 g Na_2_SO_4_ x 6 H_2_O; 0.7057 g NaHCO_3_; 0.456 5g K_2_HPO_4_ x 3 H_2_O; [40]). Both stock solutions were filled up to 1 L with double distilled water. Swelling of the foams was determined after 0, 1, 2, 4 and 24 h and the increase in diameter and length was determined as compared to the diameter and length in the dry foam (= 0 % swelling). Data represent means of 4 different samples ± standard deviation per condition.

### 3.4. Flow device

A custom-made flow device was used consisting of two parts A and B that could be connected through a screw thread leaving a sample container of 5 mm inner diameter and adjustable length (2-5 mm) by insertion of different numbers of O-rings between parts A and B [20]. Each part was manufactured in poly-methyl-methacrylate (PMMA) that allowed optical inspection of the flow chamber. To ensure non-leakiness of the chamber, fittings for the attachment of the tubing were manufactured directly attached to parts A and B, respectively. TYGON R3607 silicon tubes with the following dimensions were used: 1.62 mm outer and 0.76 mm inner diameter.

### 3.5. Determination of interconnectivity of the pores in DP-, PU- and PVA-foams

To determine the interconnectivity of the foam pores fluorescent beads of different diameter were circulated through DP-, PU- and PVA-foams in a closed-loop system (Figure 2). The adsorption of the beads on and within the foams was assessed by decreased fluorescence of the bead solution. 3 µm Fluoresbrite^TM^ YG microspheres (No. 17155, Polysciences, Inc), 20 µm Fluoresbrite^TM^ YG microspheres (No. 19096, Polysciences, Inc) and 45 µm Fluoresbrite^TM^ YG microspheres (No. 18242, Polysciences, Inc) were used for the experiments. The fluorescent beads were used either native in PBS or after pre-adsorption with bovine serum albumin. For this purpose 100 µL of the bead solution was pre-adsorbed in 1 mL 2 % bovine serum albumin (BSA, Sigma, A 9418) in PBS and incubated for 15 min, centrifuged 5 min at 10,000 rpm at room temperature. This procedure was repeated three times. The foams were pre-swollen in PBS at room temperature and cut into disks of 5 mm diameter and 5 mm length and inserted into the custom-made flow device that allowed perfusion with fluorescent beads. The flow rate was 8.1 mL/min using an Ismatec instruments pump (No. 7610-20). As the fluorescence of the circulating solution correlated with the number of fluorescent beads, two times 200 µL samples were extracted before and after 5, 20 min, 1 and 4 hours, and 1 and 3 days circulation time and transferred into white 96 well plates. The fluorescence was determined at 441 nm excitation and 486 nm emission wavelength both at 2.5 nm slit width in a PerkinElmer LS 55 Luminescence Spectrometer using the provided software Flwinlab. The numbers of the fluorescent beads in solution submitted through the foams were 8.4 x10^7^ for the 3 µm diameter beads, the 20 µm bead solution contained 284,000 beads and the 45 µm bead solution 24,950 beads, respectively. The fluorescence at the beginning of the experiments corresponded to the number of beads (for the appropriate size of beads) and has been used as a reference. At the indicated time points the actual fluorescence was determined and relative fluorescence = $\frac{actual\;\;fluorescence}{initial\;\;flluorescence}\cdot 100$ was calculated. The values represent mean values ± standard deviation of at least three individual experiments carried out in triplicate per experimental condition. All values were reduced by the value obtained for the solvent (PBS) only.

### 3.6. Morphological characterization of polymer foams

#### 3.6.1. Macroscope images

Cylinders of DP-, PU- and PVA-foams (5 mm in diameter and 5 min in length) were swollen in PBS, perfused with 3 or 20 m diameter fluorescent beads for 24 h as indicated above or left in PBS only. The foams were fixed in 4 % paraformaldehyde dissolved in PBS for 10 min at room temperature. Then they were extensively rinsed with PBS and placed on a clean glass plate. The samples were analyzed by a Leica MZ 16A macroscope using upright light and the front lens No. 1.

#### 3.6.2. Scanning electron microscopy (SEM)

Cylinders of DP-, PU- and PVA-foams (5 mm in diameter, 5 mm in length) were swollen in PBS and fixed in 4 % paraformaldehyde dissolved in PBS for 10 min and in 3 % glutaraldehyde in PBS for 30 min both at room temperature. The samples were dehydrated in a graded series of ethanol. Out of absolute ethanol the samples were dried over the critical point of CO_2_ (T_k_ = 31 °C, P_k_ = 73, 8 bar) using a critical-point dryer (CPD 030 Critical Point Dryer, Bal-Tec AG, Balzers, Liechtenstein). The samples were sputter-coated with 10 nm platinum and the images were recorded with a Zeiss SUPRA 50 VP at 1 kV and 5 kV using secondary electron signals.

## 4. Conclusions

This study demonstrates that the geometry of different NPWT dressings, even in their non-compressed form, is not the only parameter decisive for effective bead penetration but also the chemistry of the foam itself resulting in differential affinity between the beads and the polymers. In addition due to the efficient sieve-effect of the foams uptake of vacuum-removed wound components *in vivo* might occur only for short time suggesting biological mechanisms rather than materials characteristics being decisive for clinical success of NPWT in wound healing.
